# Expression of a novel carbonic anhydrase, CA XIII, in normal and neoplastic colorectal mucosa

**DOI:** 10.1186/1471-2407-5-41

**Published:** 2005-04-18

**Authors:** Laura Kummola, Jonna M Hämäläinen, Jyrki Kivelä, Antti J Kivelä, Juha Saarnio, Tuomo Karttunen, Seppo Parkkila

**Affiliations:** 1 Institute of Medical Technology, University of Tampere, and Tampere University Hospital, Tampere, Finland; 2 Institute of Dentistry, University of Helsinki and Research Institute of Military Medicine, Central Military Hospital, Helsinki, Finland; 3 Department of Surgery, University of Oulu, Oulu, Finland; 4 Department of Pathology, University of Oulu, Oulu, Finland; 5 Department of Clinical Chemistry, University of Oulu, Oulu, Finland

## Abstract

**Background:**

Carbonic anhydrase (CA) isozymes may have an important role in cancer development. Some isozymes control pH homeostasis in tumors that appears to modulate the behaviour of cancer cells. CA XIII is the newest member of the CA gene family. It is a cytosolic isozyme which is expressed in a number of normal tissues. The present study was designed to investigate CA XIII expression in prospectively collected colorectal tumor samples.

**Methods:**

Both neoplastic and normal tissue specimens were obtained from the same patients. The analyses were performed using CA XIII-specific antibodies and an immunohistochemical staining method. For comparison, the tissue sections were immunostained for other cytosolic isozymes, CA I and II.

**Results:**

The results indicated that the expression of CA XIII is down-regulated in tumor cells compared to the normal tissue. The lowest signal was detected in carcinoma samples. This pattern of expression was quite parallel for CA I and II.

**Conclusion:**

The down-regulation of cytosolic CA I, II and XIII in colorectal cancer may result from reduced levels of a common transcription factor or loss of closely linked *CA1*, *CA2 *and *CA13 *alleles on chromosome 8. Their possible role as tumor suppressors should be further evaluated.

## Background

Carbonic anhydrases (CAs) are a growing family of zinc-containing metalloenzymes that have an important role in maintaining the pH homeostasis by catalysing the reversible hydration of carbon dioxide, CO_2 _+ H_2_O ↔ HCO_3 _^- ^+ H^+^. This family includes at least twelve enzymatically active isozymes, whose subcellular localization, tissue distribution and kinetic properties differ. Five of the isozymes are cytosolic (CA I, II, III, VII and XIII), four are membrane associated (CA IV, IX, XII and XIV), two are mitochondrial (CA VA and VB) and one is a secretory form (CA VI) [1-6]. CAs contribute to several important biological functions, such as respiration, bone resorption, renal acidification, gluconeogenesis and formation of cerebrospinal fluid and gastric acid [1,7].

It is typical for solid tumors to create a pH gradient between the intracellular and extracellular compartments by acidifying the surrounding tissue to lower pH value than in adjacent normal tissue. Extracellular acidification has been associated with many tumor progression effects, such as loss of intercellular adhesion, increased metastasis and migration, increased rate of mutations and reduced DNA repair and upregulation of growth factors and proteases [4]. In order to maintain the pH gradient, tumor cells express ion transport proteins, including vacuolar H^+^-ATPase, Cl^-^/HCO_3 _^- ^exchanger and Na^+^/H^+ ^exchanger [8,9]. Previously, lactic acid has been considered a major source of acidity in tumors [4]. More recent studies have indicated, however, that carbon dioxide also makes a significant contribution to the tumor acidity, which strongly implies to the importance of CA activity in oncogenesis. This hypothesis has been indirectly supported by the recent findings that several CA inhibitors may suppress the invasion capacity of malignant cell lines [4].

CA XIII is a novel enzyme, which is expressed in several human tissues including salivary glands, kidney, small intestine, colon, uterus and testis. Modelling of CA XIII protein revealed that is a globular molecule, and it has the highest (about 60%) identity with cytosolic isozymes CA I, II and III. Therefore, CA XIII is clustered as cytosolic/intracellular isoform. In the previous report, we described the production of recombinant mouse CA XIII protein, which was used in detailed activity and inhibition studies [2,10]. CA XIII demonstrated moderate CA catalytic activity with *k*_*cat*_/*K*_*m *_of 4.3 × 10^7 ^M^-1^s^-1^, and *k*_*cat *_of 8.3 × 10^4 ^s^-1 ^at 25°C and pH 7.5 [2]. Interestingly, CA XIII showed unique inhibitory properties compared to CA I, II and IX [10]. The clinically used sulfonamides including acetazolamide, methazolamide, dichlorophenamide, and dorzolamide demonstrated potent CA XIII inhibition, with *K*_i_'s in the range of 17–23 nM. Another group of inhibitors, the sulfanilyl-derived compounds, obtained by reaction of aminosulfonamides with 4-acetamido-benzenesulfonyl chloride showed very potent CA XIII inhibitory properties, with *K*_i_'s in the range of 1.3–2.4 nM, whereas these compounds were generally much less effective as inhibitors of isozymes I, II, and IX.

Different CA isozymes have become attractive targets in studies aimed at molecular mechanisms of carcinogenesis. CA IX and XII have been clearly associated with certain types of cancer [11-21]. Also cytosolic isozymes CA I and II have been investigated in some tumors including colorectal cancer [22,23]. Since there was no previous data on CA XIII in tumors, we collected a series of colorectal neoplasias and adjacent normal tissues, and analyzed them by an immunohistochemical staining method for the expression of this novel isozyme.

## Methods

### Antibodies

The rabbit anti-mouse/human CA XIII antibody has been described in a recent article [2]. It was raised against a synthetic peptide (AC-) DGDQQSPIEIKTKEC (-COOH). The affinity and specificity of the antibody was confirmed by ELISA titre analysis, western blotting and immunohistochemistry. In immunohistochemical staining, CA XIII has shown a distinctly different distribution pattern compared to the localization of CA I or II [2]. This finding indicates that the anti-CA XIII antibody does not cross-react with CA I or II under the present staining conditions. Rabbit antisera against human CA I and II have been produced and characterized earlier [24].

### Immunohistochemistry

The normal tissue samples from the large intestine and the corresponding benign and/or malignant neoplastic samples were prospectively collected at Oulu University Hospital (Oulu, Finland). The normal samples were excised separately 20 centimeters proximally from the tumor resection site. The study was approved by the Ethics Committee of Oulu University Hospital and performed according to the guidelines of the Declaration of Helsinki.

32 distinct areas (both normal tissue and pathological lesions) of the human colorectal mucosa were examined from 12 patients. They consisted of 11 separate samples of histologically normal human colon or rectum and 17 colorectal lesions, including 6 adenomas and 11 adenocarcinomas. In 4 samples, normal tissue was found adjacent to the neoplasm and was included in the analyses. The grade of dysplasia was moderate in 3 and grave in 3 adenomas. The group of 11 malignant colorectal tumors consisted of 4 well-differentiated and 4 moderately differentiated adenocarcinomas, and 3 adenocarcinomas with a mucinous component. It should be noted that each patient had either one or more (1–3) lesions which were analyzed.

The specimens were fixed in Carnoy's fluid (absolute ethanol + chloroform + glacial acetic acid 6:3:1) for 6 hours. The samples were then dehydrated, embedded in paraffin in a vacuum oven at 58°C, and sections of 5 μm were placed on gelatine-coated microscope slides The CA isozymes were immunostained by the biotin-streptavidin complex method, employing the following steps: (a) pre-treatment of the sections with undiluted cow colostral whey (Biotop Oy, Oulu, Finland) for 30 min and rinsing in phosphate-buffered saline (PBS), (b) incubation for 1 hr with primary antibodies diluted 1:100 in 1% bovine serum albumin -PBS (BSA-PBS), (c) incubation for 1 hr with biotinylated goat anti-rabbit IgG (Zymed laboratories inc., South San Fransisco, CA) diluted 1:300 in 1% BSA-PBS, (d) incubation for 30 min with streptavidin-horseradish peroxidase (HRP) conjugate (Zymed laboratories) diluted 1:750 in PBS and (e) incubation for 2 min in DAB solution containing 4,5 mg 3,3'-diaminobenzidine tetrahydrochloride (Fluka, Buchs, Switzerland) in 7,5 ml PBS + 2,5 μl 30% H_2_O_2_. The sections were washed three times for 10 min in PBS after incubation steps b and c, and four times for 5 min in PBS after step d. All the incubations and washings were carried out at room temperature. The sections were finally mounted in Neo-Mount (Merck, Darmstadt, Germany). The stained sections were examined and photographed with Zeiss Axioskop 40 microscope (Carl Zeiss, Göttingen, Germany). The intensity of immunostaining was scored on a scale of 0 to 3 as follows: 0, no reaction; 1, weak reaction; 2, moderate reaction; 3, strong reaction.

## Results and discussion

Carbonic anhydrase XIII is a recently discovered enzyme, which is expressed in a number of different mammalian tissues. Structural modelling revealed that it is most closely related to cytosolic isozymes, CA I, II, and III with about 60% sequence identity, and therefore, this isozyme was clustered to the group of cytosolic/intracellular isoforms. Even though the previous paper described the distribution of CA XIII in several normal tissues, no data has been published to date on its expression in neoplastic tissues. To get a better insight into the changes in the expression levels that occur during the development of neoplasia, we analyzed both normal and pathological colorectal specimens from the same individuals.

**Figure 1:**
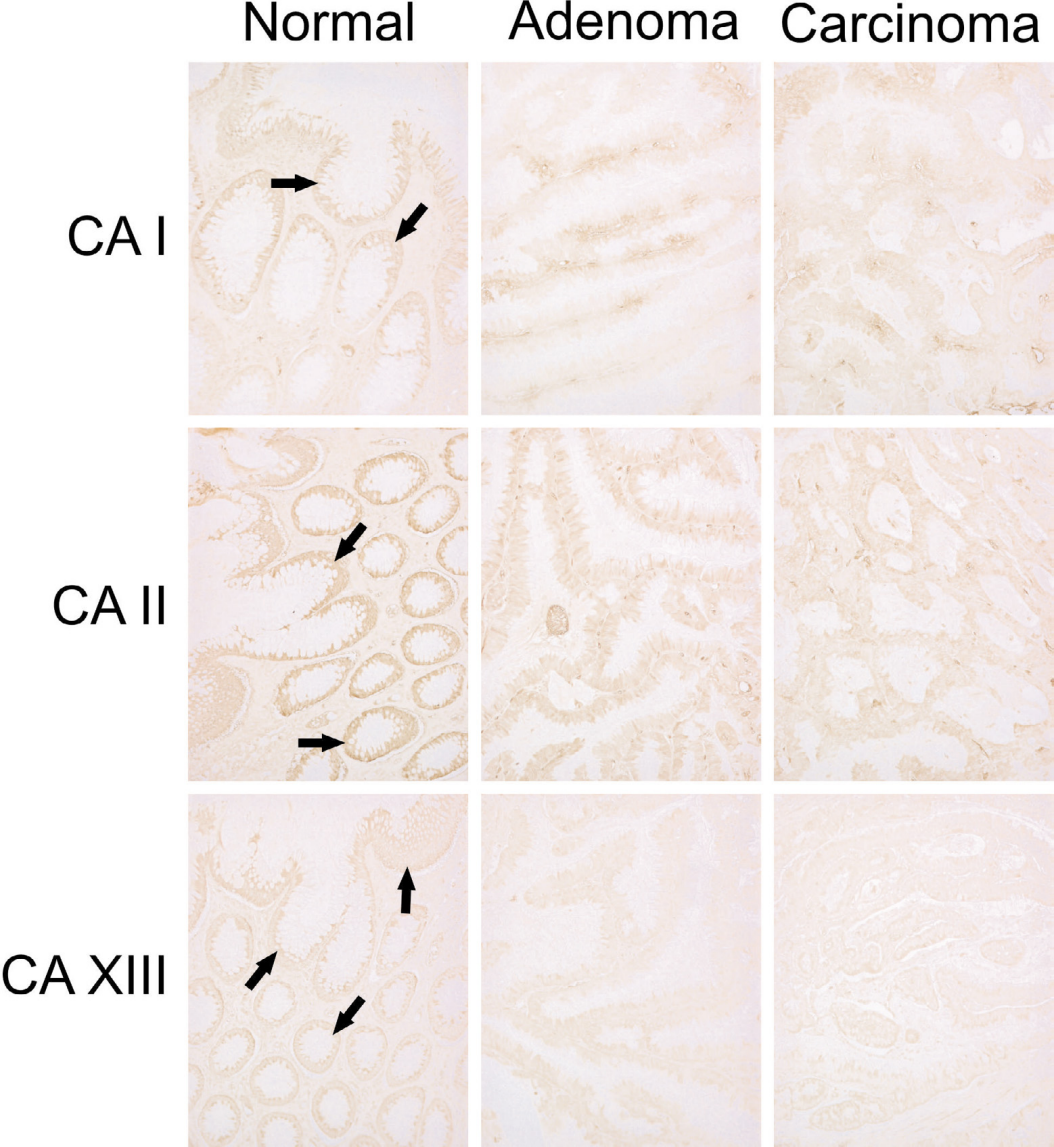
**Immunohistochemical staining of CA I, II and XIII in the normal colonic mucosa and tumors. **The samples of normal mucosa, adenoma (grave dysplasia) and well differentiated adenocarcinoma were collected from one patient. The strongest immunoreaction is located in the enterocytes of the normal mucosa (arrows). Original magnification × 200.

**Figure 2:**
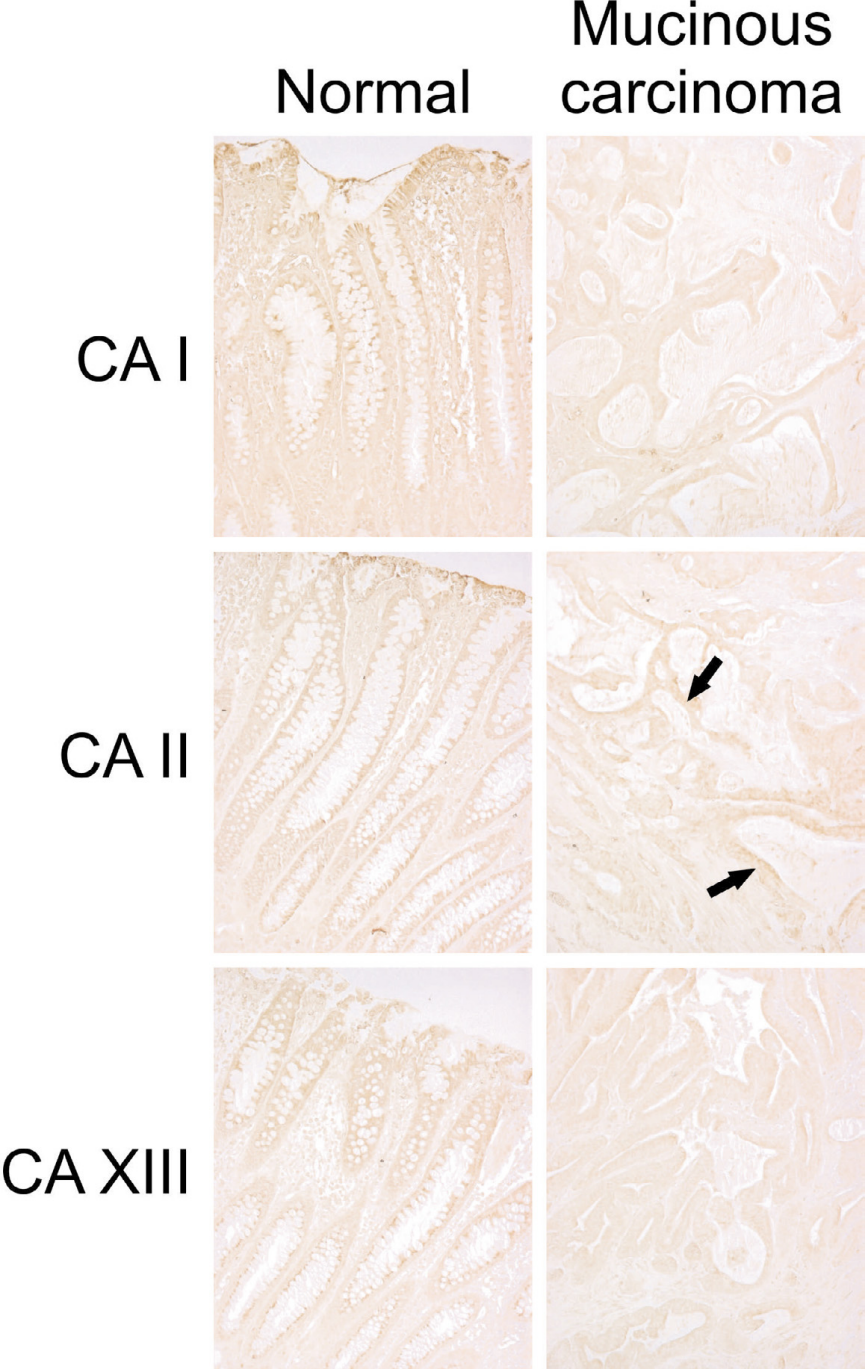
**CA expression in the normal colonic mucosa and adenocarcinoma with a mucinous component. **CA II staining intensity remained at a relatively high level in the carcinoma sample (arrows), whereas the intensities for other isozymes were decreased compared to the normal epithelium.

**Figure 3:**
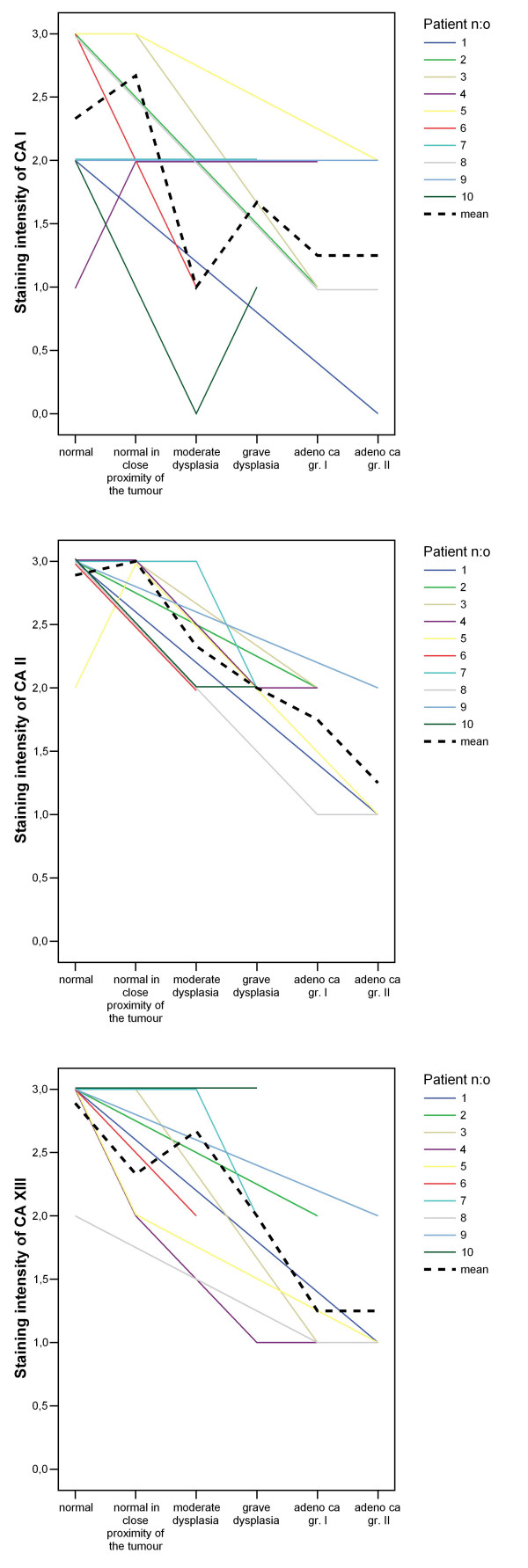
**A graphic summary of CA immunostaining in the normal colorectal mucosa and tumor samples. **The coloured lines represent the staining indices in individual patients. The dash lines indicate the mean values of staining intensity.

Figures 1 and 2 show some images of CA XIII immunostaining in the colorectal tumors and normal mucosa. Reactions for CA I and II are shown for comparison. The immunostaining scoring results are shown in Figure 3. The results indicated that CA XIII immunoreactions generally follow a similar pattern with CA I and II, i.e. the signals became weaker along with increasing dysplasia and malignancy grades. There were a few exceptions, and particularly, CA II expression was relatively high in adenocarcinomas with a mucinous component, while CA I and XIII were decreased like in other adenocarcinomas (Fig. 2).

Previous studies have indicated that CA IX and XII are overexpressed in certain tumors, including colorectal adenomas and carcinomas [18,23]. They have been functionally linked to extracellular acidification in tumors that appears to regulate the invasive behaviour of tumor cells. Consequently, expression of CA IX has been associated with poor prognosis of some malignancies [19,20,25,26]. Based on the present and previous results [23], cytosolic isozymes I, II, and XIII do not probably play a significant role in the regulation of pH in tumors. Instead, they may be important factors for the normal intestinal physiology. This suggestion raises an important question why at least three cytosolic isozymes are expressed in the normal colorectal mucosa. Even though we cannot answer to this question at this stage, it is of interest that these CAs represent isoforms with different kinetic or inhibitory properties. CA II exhibits the highest and CA I and CA XIII moderate carbon dioxide hydratase activities [2]. Because of different biochemical characteristics, it is unlikely that all these isozymes could contribute to the same pH regulation process. If this was the case, one could suggest that the enzymes with low or moderate activities should represent some sort of "junk" proteins in the proteome. However, there are other possible explanations which could be considered more sound in a biological system. First, several CA isozymes have emerged during evolution, because CA enzymatic activity is required for very fundamental reactions in the body. If one isozyme was functionally defective, the others could compensate the missing one. These compensatory mechanisms would partly explain why different CA deficiencies typically produce no or only mild phenotypic changes [27-29].

Another possible role of cytosolic CAs could be linked to a hypothetical tumor suppressor function. It is notable that *CA1*, *CA2 *and *CA13 *genes are closely linked locating in the q arm of chromosome 8. Simultaneous loss of expression could result from a deletion or point mutations within regulatory regions or alleles specifying these genes. The down-regulation could also result from reduced levels of a common transcription factor. Regardless of the mechanism, three cytosolic CA isozymes are affected in colorectal tumors, and it would be tempting to propose that normal CA I, II and/or XIII proteins could have a tumor suppressor function in epithelial cells, and deletion of those alleles could be an important factor in the cascade of events contributing to colorectal carcinogenesis. Even though colorectal cancer has been associated with gain of chromosome 8q in a number of previous studies, and no losses have been reported [30-33], there is some evidence that 8q21 contains a yet unidentified tumor suppressor gene [34,35]. However, larger sets of tumors will be required to define explicitly, whether these *CA *genes are involved in genetic alterations of colorectal tumors.

## Conclusion

CA XIII expression was clearly decreased in colorectal tumors compared to the normal tissue in a pattern similar to CA I and II. Loss of expression of closely linked *CA1*, *CA2 *and *CA13 *genes may result from reduced levels of a transcription factor or loss of alleles specifying these genes.

## Competing interests

The author(s) declare that they have no competing interests.

## Authors' contributions

All authors participated in the design of the study. JS, TK and AJK collected the tissue samples. LK and SP drafted the manuscript. JMH and SP characterized the antibodies. LK performed the immunohistochemical staining. LK, JK and SP analyzed the staining results. All authors read, modified and approved the final manuscript.

## Pre-publication history

The pre-publication history for this paper can be accessed here:
